# An open-hardware platform for conservation of amphibian genetic resources based on gelatin capsules

**DOI:** 10.1016/j.isci.2026.117103

**Published:** 2026-08-10

**Authors:** Thaiza Rodrigues de Freitas, Danilo Pedro Streit, Elise Harmon, Nathalia dos Santos Teixeira, Jhony Lisboa Benato, Thales de Souza França, Jack Cushman Koch, Terrence Robert Tiersch

**Affiliations:** 1 Aquatic Germplasm and Genetic Resources Center, Louisiana State University Agricultural Center, Baton Rouge, LA 70820, USA; 2 AQUAM Research Group, Animal Science Research Program, Federal University of Rio Grande do Sul, 7712 Bento Goncalves Avenue, Porto Alegre, Rio Grande do Sul 91540-000, Brazil; 3 Veterinary Science Research Program, Federal University of Rio Grande do Sul, 9090 Bento Goncalves Avenue, Porto Alegre, Rio Grande do Sul 91540-000, Brazil; 4 Universitat Politècnica de València, Instituto de Ciencia y Tecnologia Animal, Camí de Vera, s/n, Building 7G, 46022 Valencia, Comunidad Valenciana, Spain; 5 Aquatic Germplasm and Genetic Resources – Center of Research Excellence, Louisiana State University Agricultural Center, Baton Rouge, LA 70820, USA

**Keywords:** open hardware, gelatin capsule, *Xenopus laevis*, germplasm repository development, frog

## Abstract

Amphibian biodiversity is declining worldwide, and accessible cryopreservation tools are needed to support germplasm repositories, especially in low-resource settings. We developed a platform that integrates 3D-printed open-hardware components with gelatin capsules as containers for amphibian sperm cryopreservation, using *Xenopus laevis* as a model. We compared a 3D-printed platform (CryoKit) to a computer-controlled freezer and evaluated capsule thawing strategies. The CryoKit reached −80 °C faster than the computer-controlled freezer (4.0 vs. 6.6 min) and produced similar post-thaw viability (54% vs. 50%), forward motility (11% vs. 9%), and twitching movement (21% vs. 22%). Capsules showed comparable thermal performance to straws (5.5 vs. 4.3 min), and produced similar viability (37% vs. 38%), forward motility (5% vs. 9%), twitching (23% vs. 20%), and fertilization (16% vs. 37%). The dry-thawing method prevented capsule dissolution while preserving fertilization capacity. Our open-hardware designs and practical thawing protocol provide an accessible, reproducible approach to amphibian sperm cryopreservation suitable for resource-limited settings.

## Highlights

• Open hardware system enabled amphibian sperm cryopreservation in gelatin capsules
• 3D-printed platform achieved comparable performance to a computer-controlled freezer
• Gelatin capsules provided a practical alternative to conventional French straws
• Dry thawing preserves sperm quality without capsule dissolution

## Graphical abstract

**Figure:**
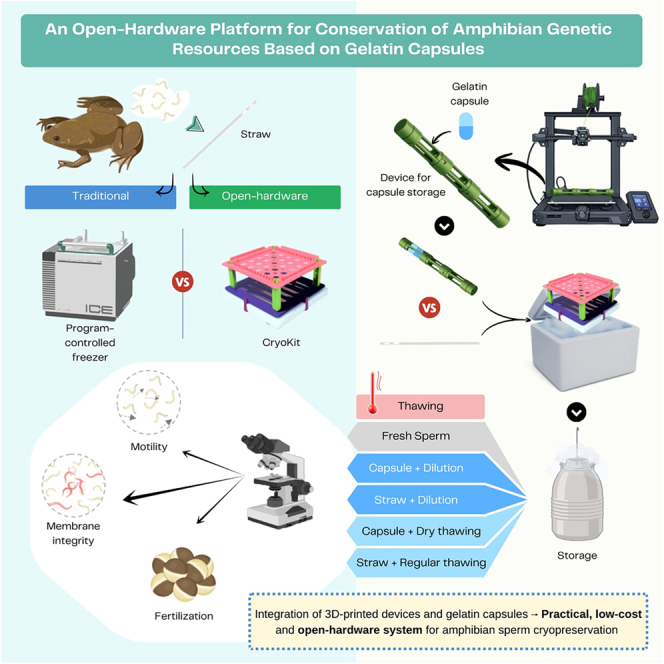

## Teaser

Reproductive medicine; environmental science; biological sciences

## Introduction

Amphibians have been facing a global population decline, with at least 41% of species currently threatened worldwide.^1^ This decline is unprecedented in speed and magnitude, having occurred largely within the past four decades.^2^ Major drivers include habitat degradation and fragmentation,^3^ climate change,^4^ and spread of diseases like chytridiomycosis.^5^^,^^6^ Amphibian physiology, including permeable skin and limited dispersal ability, makes them particularly vulnerable to rapid environmental changes and disease.^7^ In light of this crisis, captive breeding programs (CBPs) have become essential in protecting imperiled species.^8^ These programs aim to create assurance colonies which can be supplemented by germplasm repositories.^9^ Incorporating sperm cryopreservation into amphibian CBPs can reduce monetary costs and improve retention of genetic diversity.^9^^,^^10^ In addition, cryopreserved sperm can remain frozen for years offering a reliable method to generate future populations and recover lost genetic lineages.^11^

Cryopreservation is considered the most cost-effective option for maintaining genetic resources, as it can reduce costs related to the maintenance of animals, facility operation, and personnel.^12^ However, despite its potential, it typically comes with high upfront costs,^13^ particularly due to the price of equipment such as programmable freezers which can range from US$ 15,000 to US$ 60,000.^14^ The cost of liquid nitrogen tanks, liquid nitrogen, laboratory materials (e.g., vials, straws, and chemicals), facilities, and skilled professionals also significantly increase expenses. While cryopreservation remains an effective solution, it continues to be a challenge for many, especially in low- and middle-income nations where the establishment of germplasm repositories is not considered a development priority^15^ but could provide benefits from implementation. Overall, there are limited research, investment, and conservation efforts for most amphibian species.^16^ Therefore, it is necessary to explore and adopt more accessible and low-cost options to ensure effective preservation of gene pools without placing a heavy financial burden on institutions and developing countries.

In addition to the high cost of commercially available freezing devices, non-standardizable custom devices (e.g., do-it-yourself) represent a significant barrier to community sharing of resources and quality management,^14^ often preventing the reproducibility of protocols. In this context, the use of three-dimensional (3D) printed open-hardware devices has been integrated into safeguarding genetic resources of aquatic species^17^ and in development of germplasm repositories. Two examples of this are the positional cooling platform (PCPD, known as “CryoKit”)^14^ and the shipping dewar positional cooling device (SDPCD, known as “Cajun Ejector”).^18^ Each of these consists of several 3D printed parts that interconnect to create a functional device designed to provide reproducible cooling for less than US$ 10 material cost. This approach has contributed to creation of a genetic resource community initiative that can promote safeguarding and exchange of cryopreserved materials. Open technologies can increase global accessibility to standardizable devices that can also be customized for different species and applications. This can enable a decentralized (aggregate-throughput) model for widespread development of germplasm repositories.^19^

The container for cryopreservation must be considered early in process development as the container chosen can constrain the downstream technologies and processing options. Gelatin capsule as cryopreservation container, have been employed in cryopreservation of fish sperm^20^^,^^21^ and gonadal tissue^22^ offering an affordable alternative to traditional cryopreservation containers like cryovials (polypropylene) and French straws (polyvinyl chloride). While these traditional cryopreservation containers are commonly used in cryopreservation,^23^ they have relatively higher market prices and can have limited availability in certain countries.^20^ This is especially true in countries without a strong cattle industry, which typically drives the market for cryopreservation resources.^24^ In contrast, gelatin capsules, most commonly used in medicine encapsulation, are more accessible with lower cost and widespread availability^20^^,^^21^ making them a promising option in low-resources scenarios. However, gelatin capsules remain inefficient in processing, storage, and use without supporting tools and systems. Alone, the drawbacks of gelatin capsules would outweigh their advantages, especially with increasing scale of use. However, combination with open-hardware approaches could provide efficient, inexpensive, and scalable platforms to support germplasm repository development.

Therefore, the goal of this study was to improve accessibility for amphibian sperm cryopreservation by developing a new open-hardware platform for handling, freezing, storage, and use with gelatin capsules, using *Xenopus laevis* sperm as a case study. Our objectives were to: (1) design a 3D-printed rack for sample loading in gelatin capsules and a holder for processing and storage of the gelatin capsules; (2) assess the cooling performance and post-thaw sperm quality when conducting cryopreservation cooling using a 3D printed open-hardware device (CryoKit) versus a standard computer-controlled freezer, and (3) validate the capsule and 3D-printed modular holder system by comparing *X. laevis* sperm cryopreserved in gelatin capsules with sperm cryopreserved in French straws, including evaluation of a dry-thawing method to prevent capsule dissolution and enable control of sperm dilution.

## Results

### Capsule device development

**Figure 1:**
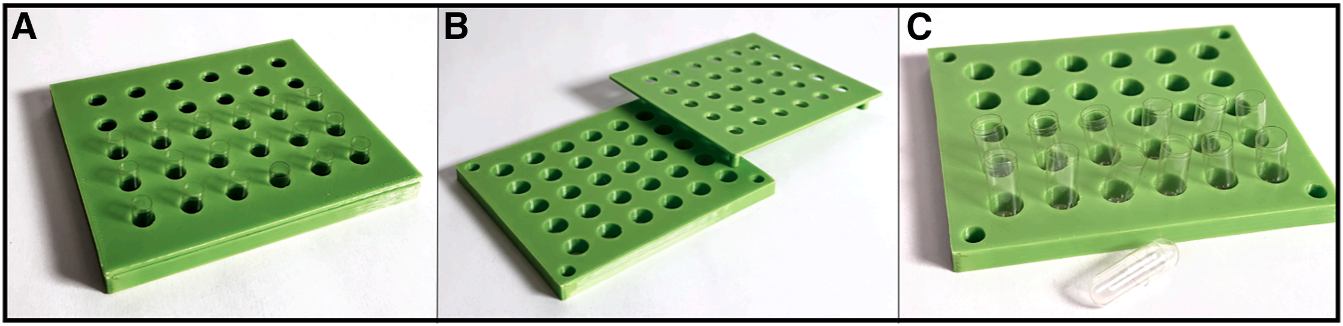
Three-dimensional printed open-hardware rack developed to support gelatin capsules during sample loading (A) Assembled rack used for size 3 gelatin capsules, showing the upper part with smaller holes for secure fitting. (B) Rack with the upper part removed. (C) Base part used for size 0 gelatin capsules, with larger holes.

A dedicated filling rack was developed to facilitate loading gelatin capsules. The rack was designed to hold up to 30 gelatin capsules in an upright, stable position during sample loading (Figure 1). It consisted of two interlocking parts, allowing use with gelatin capsules of different sizes. When assembled, it served as a support for size 3 capsules (Figure 1A), with smaller holes on the upper piece to securely hold the capsules. When the upper piece was removed (Figure 1B), the base could be used for size 0 capsules, which fit into the larger holes (Figure 1C). The device measured 91 × 78 × 8.5 mm (length × width × depth) and had an estimated material cost of ∼US$ 0.84 per unit. In practice, the rack provided stable and secure positioning during pipetting.

**Figure 2:**
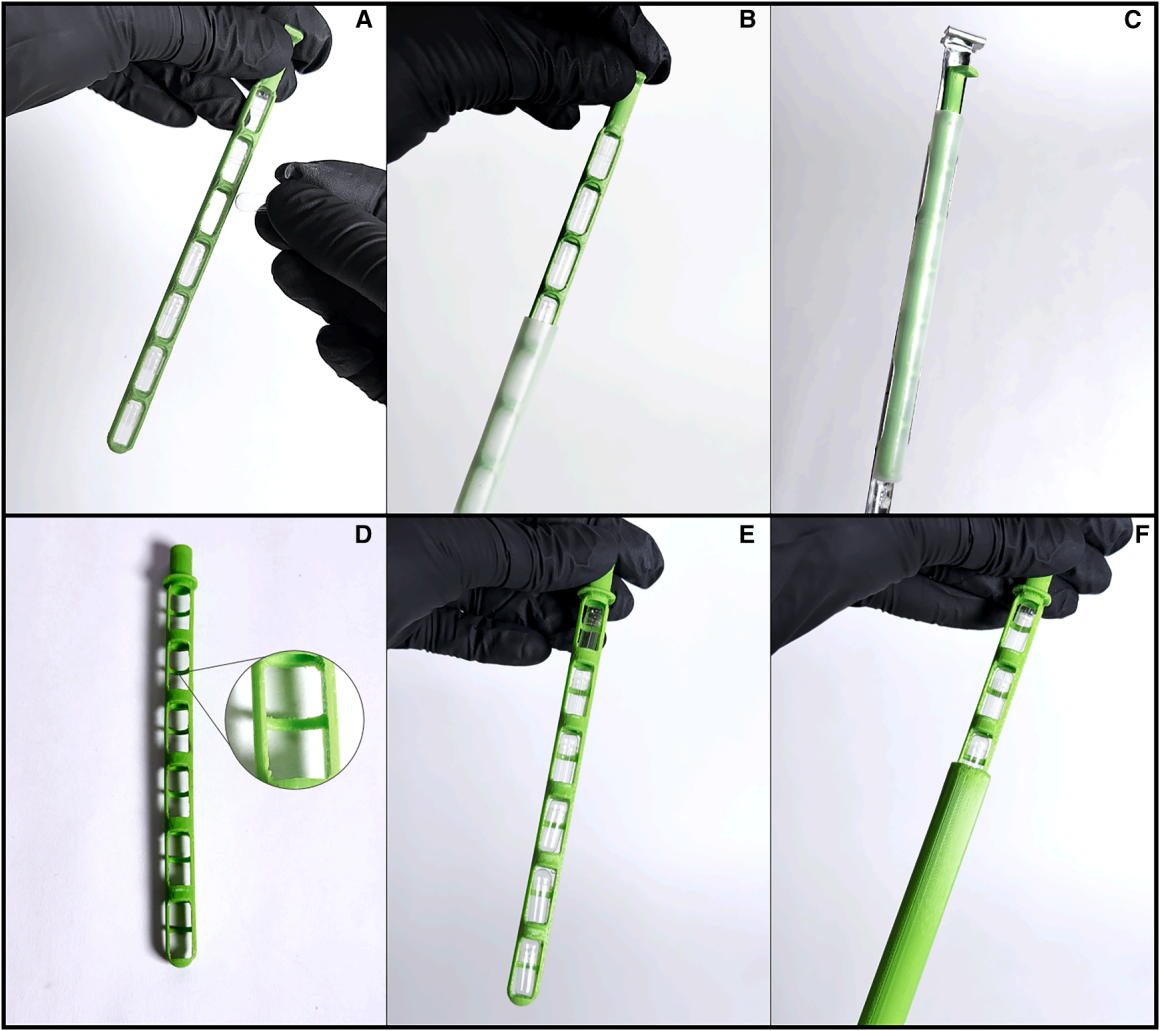
Sequence of prototype versions developed of the open-hardware system for cryopreservation processing using gelatin capsules (A) Capsule arrangement in prototype V1.0. (B) Holder positioned inside a 10-mm goblet. (C) Assembly attached to an aluminum cryo-cane. (D) Prototype V2.0 featuring enlarged cavities and a rear support for gelatin capsule retention; inset shows a close-up of the capsule compartment highlighting the back support. (E) Gelatin capsule arrangement in prototype V2.0. (F) Complementary storage tube developed to accommodate the larger V2.0. holder.

A series of prototypes were developed and tested to address secure handling, freezing, and storage of gelatin capsules. Work of this type typically involves balancing various interacting parameters to achieve a desired outcome. For example, the first prototype (V1.0; Figures 2A–2C) was designed to securely hold six size-03 gelatin capsules (Figure 2A) and to fit inside a 10-mm visotube (Figure 2B), allowing attachment to an aluminum cryo-cane (Figure 2C). During liquid nitrogen immersion tests using mock samples, the gelatin capsules expanded upon freezing and became tightly lodged in the support; removal was not possible without damaging the capsule or the holder. A revision (V1.1) which increased the internal clearances did not fully correct the problem: gelatin capsules still became stuck after freezing.

To prevent gelatin capsules from becoming stuck, prototype V2.0 was designed with larger cavities and a back support for improved retention during handling (Figures 2D and 2E). The increased size meant the unit no longer fit within a 10-mm visotube, so we also designed a complementary storage tube to house the larger holder (Figure 2F). Although V2.0 solved the expansion problem, immersion trials revealed another issue: gelatin capsules were not retained firmly enough during rapid liquid nitrogen submersion and occasionally dislodged from the holder.

**Figure 3:**
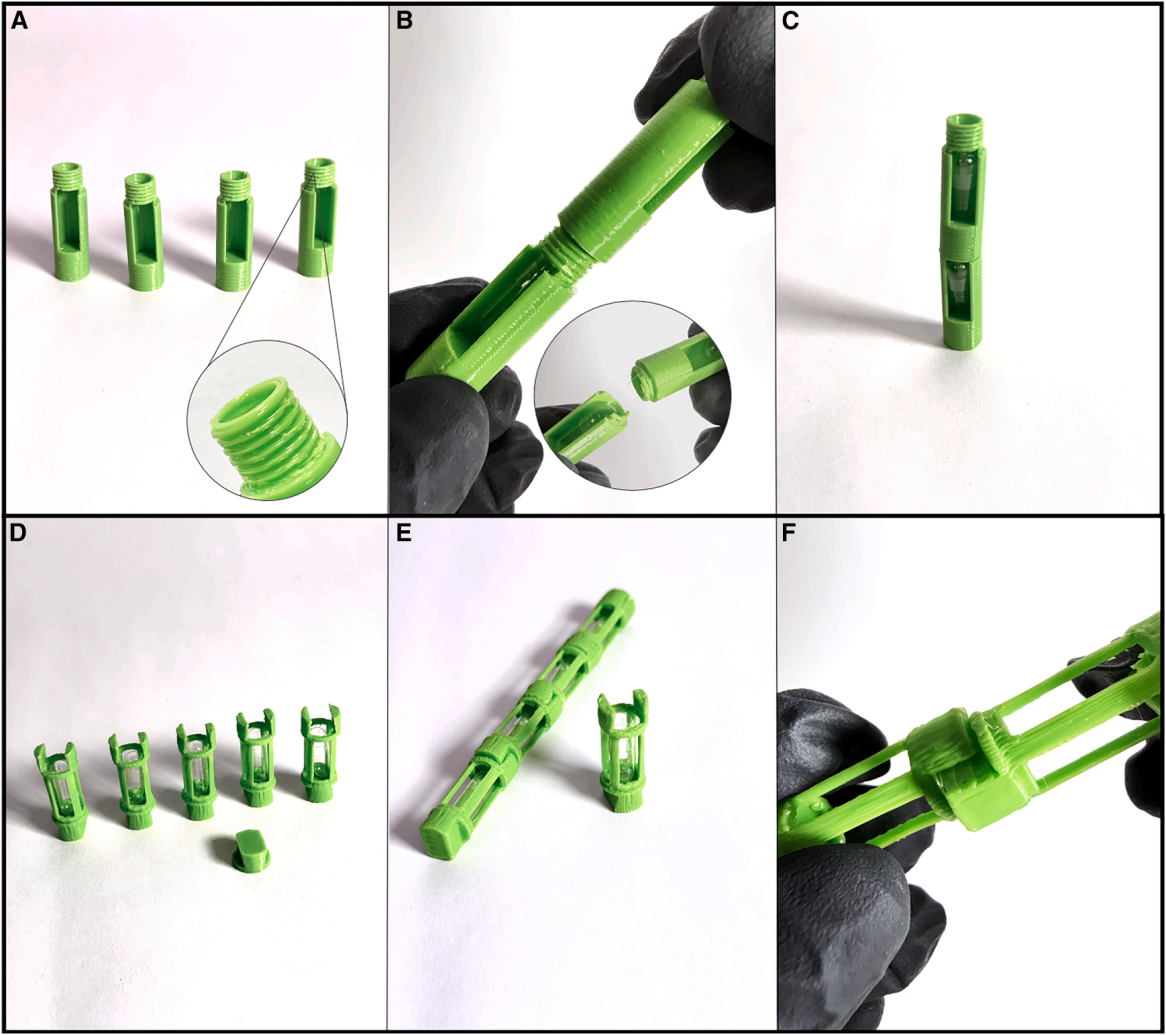
Development of the three-dimensional printed modular open-hardware for cryopreservation processing using gelatin capsules (A–C) Initial modular design (V3.0). (A) Individual modular capsule holders (V3.0); inset shows the threaded connection. (B) Assembly of the modules; inset highlights a broken thread. (C) Fully connected modular holders (V3.0). (D–F) Final modular design (V3.1). (D) Individual modular capsule holders (V3.1). (E) Connected modular holders. (F) Snap-fit connection of the V3.1 modules.

To allow individual protection and retrieval of samples, we moved to a modular design (V3.0) in which each module acted as a sleeve for a single capsule and the modules could be concatenated together (Figure 3). The first modular design used a threaded connection to join units (Figures 3A–3C); however, the threaded assembly proved difficult to handle with gloved hands, the enclosed geometry reduced heat transfer during freezing, and the threads were prone to breaking (Figure 3B), revealing a weakness of the design. Based on these observations we produced a final design (V3.1), an open, snap-fit sleeve that allowed simple insertion and removal without threads while preserving airflow and thermal exchange (Figures 3D–3F).

Each V3.1 module measured 30.2 × 11 × 11 mm and was closed with a dedicated cap with dimensions of 11 × 11 × 6.4 mm. The modules could be joined into compact stacks for organized storage and permitted retrieval of single gelatin capsules from cryogenic storage with minimal disturbance to the other samples. The open-hardware modular set (five modules plus caps) had an estimated material cost of ∼US$ 0.18. In functional testing by three PhD. students, V3.1 reliably retained gelatin capsules during liquid nitrogen handling and allowed straightforward retrieval after freezing.

A comparison of costs, throughput, and accessibility across all components and systems evaluated in this study is presented in Table 1. The CryoKit had a total material cost of <US$ 10, whereas commercial computer-controlled freezers ranged from US$ 15,000 to US$ 60,000. The CryoKit accommodated as many as 15 straws per run, while computer-controlled freezers enable batch processing of hundreds of straws per cycle. Throughput ranged from low for the CryoKit, which is intended for small-scale use and operates without electricity, too high for computer-controlled freezers, which provide programmable batch cooling but require stable electrical infrastructure and maintenance.

**Table 1:** Comparison of components and systems for sperm cryopreservation using gelatin capsules and French straws

	Unit cost (approx.)	Reusable	Throughput	Accessibility
CryoKit (open-hardware cooling platform)	< US$ 10 material cost	yes	low	3D-printable; open-hardware; no electricity required; reproducible geometry; suitable for field or low-resource settings
Computer-controlled freezer (IceCube® 14M)	US$ 15,000–60,000	yes	high	commercial equipment; requires electricity and technical maintenance; limited access in low-resource settings
Gelatin capsules	∼US$ 0.02–0.05	no	low	widely available in pharmacies and online retailers worldwide; no specialized supplier required
PVC French straws (0.25 mL)	∼US$ 0.10–0.30	no	low (individual manual filling) to high (automatic straw-filling and sealing machines)	linked to livestock reproduction industry; limited availability in regions without an established cattle sector
Capsule filling rack (open hardware)	∼US$ 0.84/unit	yes	N/A	3D-printable; open-hardware design files freely available (NIH 3D); requires basic FFF printer
3D-printed modular holder/capsule support (V3.1)	∼US$ 0.18/set of 5 modules	yes	low	3D-printable; open-hardware design files freely available (NIH 3D); requires basic FFF printer
Cryo-canes & goblets (straw storage)	∼US$ 0.50–2.00/unit	yes	moderate	linked to livestock reproduction industry; limited availability in regions without an established cattle sector

The capsule filling rack (∼US$ 0.84 per unit) and the 3D-printed modular holder (∼US$ 0.18 per set of five modules) are reusable and functionally comparable to cryo-canes and goblets (∼US$ 0.50–2.00 per unit). Gelatin capsules showed a comparable unit cost (∼US$ 0.02–0.05) relative to 0.25-mL French straws (∼US$ 0.10–0.30). Gelatin capsules are widely available through pharmacies and online retailers, whereas PVC straws, cryo-canes, and goblets depend on specialized suppliers linked to the livestock reproduction sector. All open-hardware components are 3D-printable using freely available files (NIH 3D Print Exchange) and a standard fused filament fabrication (FFF) printer.

### Sperm cryopreservation with different cooling devices

**Figure 4:**
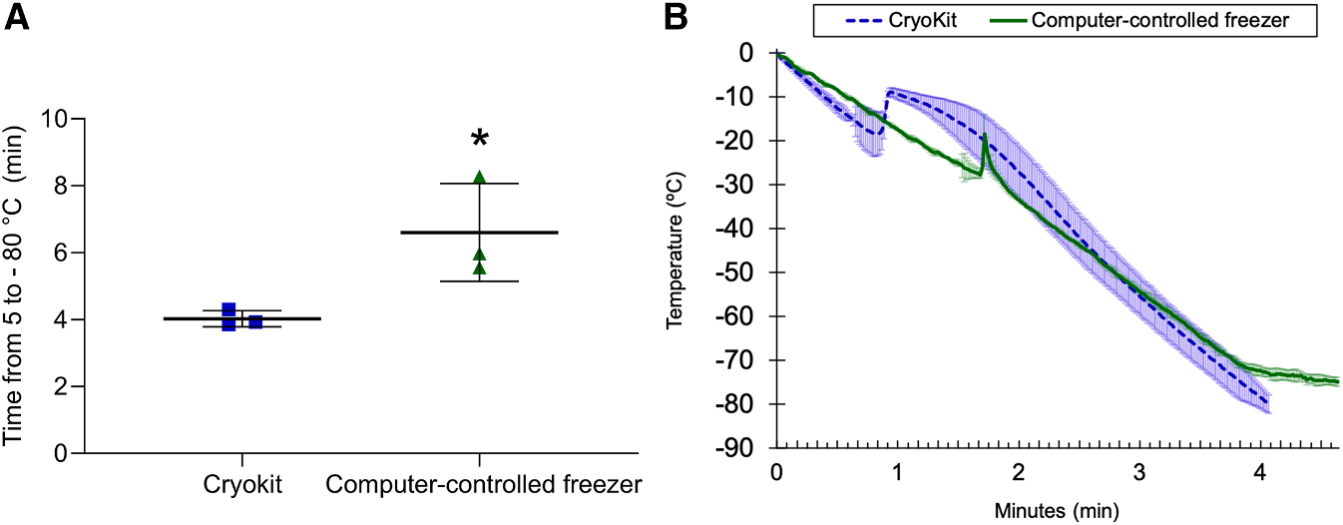
Cooling curve with different cooling devices (A) Time from 5 to −80 °C of the samples. (*p* = 0.0395, *n* = 3). (B) Sample cooling curve plot (*n* = 3); shading around each curve represents the standard deviation (SD). Asterisk indicates difference between the treatments by unpaired *t* test. Data are presented as mean ± SD (*n* = 3).

Samples cooled using the Cryokit reached −80 °C in a shorter time (4.0 ± 0.2 min) compared to those cooled in the computer-controlled freezer (6.6 ± 1.5 min; Hedges’ g = 1.94; *p* = 0.0395) (Figures 4A and 4B).

**Figure 5:**
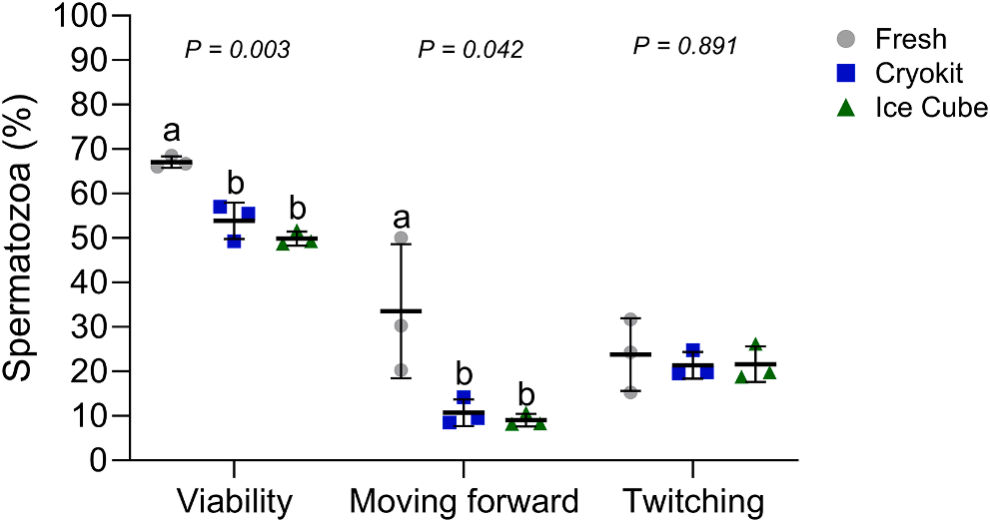
Evaluation of cryopreserved *X. laevis* sperm with different cooling devices Sperm viability (*p* = 0.003), moving forward (*p* = 0.042), twitching movement (*p* = 0.891). Different letters indicate differences among the treatments within each measured attribute (ANOVA followed by Tukey’s *post-hoc* test). Data presented as mean ± SD (*n* = 3).

The viability of the samples cryopreserved using the Cryokit (54% ± 4%) did not differ from those cryopreserved with the computer-controlled freezer (50% ± 1.6%; Hedges’ g = 1.05). However, cryopreservation reduced sperm viability compared to fresh samples (67% ± 3%), for the Cryokit (54% ± 4%; Hedges’ g = 2.94) and computer-controlled freezer (50% ± 1.6%; Hedges’ g = 5.64; *p* = 0.003) (Figure 5).

The thawed samples showed a lower percentage of forward-moving sperm compared to fresh samples (34% ± 15%) for the Cryokit (11% ± 3%; Hedges’ g = 1.70) and computer-controlled freezer (9% ± 1.4%; Hedges’ g = 1.88; *p* = 0.042). However, no differences were observed between the cooling devices (11% ± 3% vs. 9% ± 1.4%; Hedges’ g = 0.68). No differences were observed in twitching movements among treatments (Fresh: 23.8% ± 8.2%, Cryokit: 21.4% ± 3%, computer-controlled freezer: 21.6% ± 4%; *p* = 0.891) (Cryokit vs. fresh: Hedges’ g = 0.31; computer-controlled freezer vs. fresh: Hedges’ g = 0.27; Cryokit vs. computer-controlled freezer: Hedges’ g = 0.05).

### Evaluation of gelatin capsules for sperm cryopreservation

**Figure 6:**
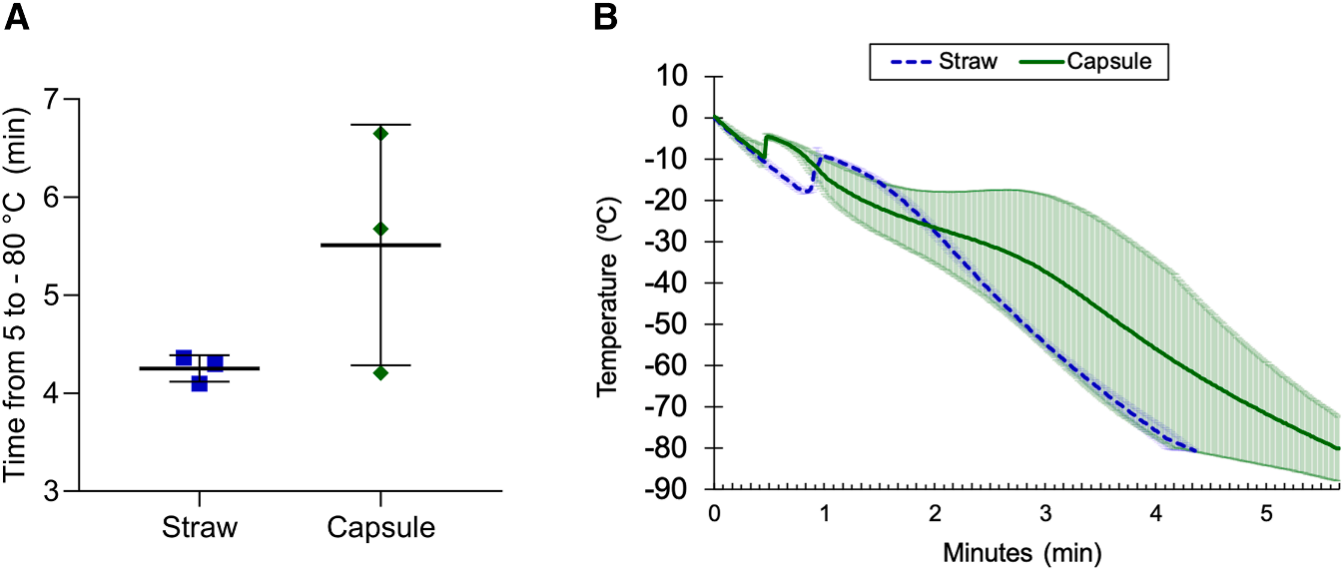
Cooling curves in different sperm cryopreservation containers (A) Time from 5 to −80 °C of the samples (*p* = 0.152, *n* = 3). (B) Sample cooling curve plot (*n* = 3); shading around each curve represents the standard deviation (SD). Data presented as mean ± SD.

There was no difference in the time required to reach −80 °C between samples cooled in gelatin capsules (5.5 ± 1.2 min) and those cooled in straws (4.3 ± 0.1 min; Hedges’ g = 1.13; *p* = 0.152) (Figure 6).

**Figure 7:**
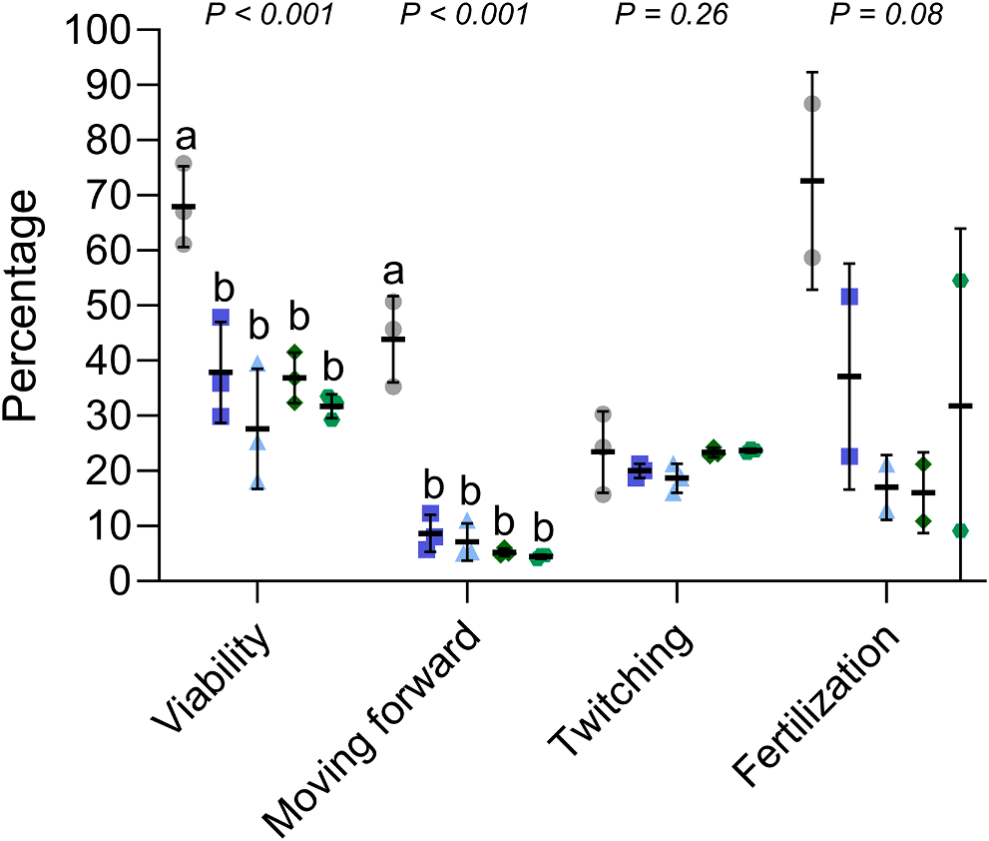
Evaluation of gelatin capsules for cryopreservation of *X. laevis* sperm and dilution during thawing Sperm viability (*p* < 0.001, *n* = 3), moving forward (*p* < 0.001, *n* = 3), twitching movement (*p* = 0.26, *n* = 3) and fertilization (*p* = 0.08, *n* = 3) were assessed. Different letters indicate differences among the treatments within quality measurements (ANOVA followed by Tukey’s *post-hoc* test). Data presented as mean ± SD.

The viability of fresh sperm samples (68.0% ± 7.4%) was higher than that observed in all cryopreserved treatments (*p* < 0.001) (Figure 7), including straw (37.9% ± 9.2%; Hedges’ g = 2.88), straw diluted after thawing (27.6% ± 0.9%; Hedges’ g = 6.13), capsule dry thawed (36.9% ± 4.6%; Hedges’ g = 4.04), and capsule diluted during thawing (31.7% ± 2.2%; Hedges’ g = 5.32). No differences were observed among cryopreserved treatments.

Fresh samples showed a higher percentage of moving forward cells (43.9% ± 7.8%) compared with all cryopreserved treatments (*p* < 0.001) (Figure 7), including straw (8.7% ± 3.4%; Hedges’ g = 4.95), straw diluted after thawing (7.1% ± 3.4%; Hedges’ g = 5.26), capsule dry thawed (5.2% ± 0.7%; Hedges’ g = 6.91), and capsule diluted during thawing (4.4% ± 0.4%; Hedges’ g = 7.10). No differences were detected among cryopreserved treatments.

No differences were observed among treatments in the percentage of cells exhibiting twitching movement (*p* = 0.26) (Figure 7), including fresh samples (23.0% ± 7.4%), straw (20.0% ± 1.3%; Hedges’ g = 0.54), straw diluted after thawing (18.7% ± 2.7%; Hedges’ g = 0.72), capsule dry thawed (23.3% ± 0.9%; Hedges’ g = 0.05), and capsule diluted during thawing (23.7% ± 0.3%; Hedges’ g = 0.12).

No differences were observed in fertilization capacity among fresh samples (72.6% ± 19.7%), and all cryopreserved treatments, including dry thawed gelatin capsules (16.0 ± 7.3%; Hedges’ g = 2.18), gelatin capsules diluted during thawing (31.8% ± 32.1%; Hedges’ g = 0.87), diluted straws (17.0% ± 5.9%; Hedges’ g = 2.18), and straws (37.1% ± 20.5%; Hedges’ g = 1.01; *p* = 0.08) (Figure 7).

## Discussion

Sperm cryopreservation is vital for preserving amphibian genetic diversity, yet the establishment of repositories in resource-limited contexts is often hindered by the high cost of materials and equipment.^13^^,^^14^ This study presented co-development of an open-hardware approach to facilitate the use of gelatin capsules as a practical container for cryopreservation. We demonstrate that this type of 3D-printed system can provide a low-cost and accessible platform for the cryopreservation of *X. laevis* sperm with potential for other amphibians.

### Capsule device development

In the pharmaceutical industry, several types of capsule-filling racks are available, some capable of loading hundreds or even thousands of gelatin capsules at once, usually costing from US$ 20 to several hundred dollars. However, those systems are designed for filling with dry powders. In this study, a specific rack was developed to hold gelatin capsules upright and stable during filling with liquid. The final version proved to be efficient, easy to use, with an estimated material cost of US$ 0.84 per unit making it an affordable and practical option that can be fabricated in any facility equipped with a basic 3D printer (∼US$ 200).

The use of gelatin capsules as containers for cryopreservation is a recent technique, and before the present study, gelatin capsules were stored in an improvised manner using 10-mm visotube (four size 0 gelatin capsules per visotube) covered with cotton and attached to an aluminum cryo-cane.^20^^,^^21^ This left the gelatin capsules, which are sensitive to abrupt temperature changes, relatively exposed, requiring careful handling.^22^ To address these problems, this study developed a 3D printed modular system which simplified and standardized handling, and enhanced sample stability and organization. This open hardware incorporated the ability to include a single capsule in each module to identify samples individually. This addresses one of the greatest challenges with gelatin capsules because it is not possible to write or print identifications directly on them without specialized printing technology (capsule printers typically cost between US$ 1,500 and US$ 40,000).

In terms of per-sample cryopreservation cost, both containers (gelatin capsules and conventional 0.25-mL French straws) are comparable when the full workflow is considered. The capsule storage support (∼US$ 0.84 per unit) and the 3D-printed modular system (∼US$ 0.18 per set of five modules) are reusable, similarly to cryo-canes and goblets used for straw storage. The unit cost of gelatin capsules (∼US$ 0.02–0.05) is slightly lower than that of straws (∼US$ 0.10–0.30); however, the main advantage of gelatin capsules is not cost reduction but accessibility. Gelatin capsules are widely available in pharmacies and commercial suppliers worldwide, whereas straws are strongly linked to the animal reproduction industry and can be difficult to obtain in regions without an established livestock sector. This makes gelatin capsules more universally accessible.

### Use of model organisms

The use of model organisms provides a valuable strategy for developing and refining biotechnologies for conservation of imperiled species, such as sperm cryopreservation. This approach allows testing and optimization of protocols before application to species of concern.^9^ This strategy reduces risks, costs, and ethical concerns while improving the efficiency of conservation efforts.^9^^,^^10^ Among amphibians, *Xenopus* species serve as well-established models,^25^ offering a practical alternative for preliminary research. Importantly, the transferable aspect of this work is the technological platform rather than species-specific reproductive traits. The workflow for loading, freezing, storing, transporting, and dry-thawing gelatin capsules can be adapted for use with other amphibian species, provided that species-specific cryopreservation parameters. Therefore, the knowledge and techniques gained from *X. laevis* provide a transferable framework that can be adapted for the development of assisted reproductive technologies for imperiled species, and should be considered for application across aquatic species in general.^26^

### Sperm cryopreservation with different cooling devices

Sperm cryopreserved using the 3D printed CryoKit produced post-thaw viability and motility comparable to samples cryopreserved using the computer-controlled freezer (IceCube). Previous work established a cryopreservation protocol for *X. laevis* sperm using a computer-controlled freezer.^27^ Here, we demonstrate that a simpler and more accessible cooling system can reproduce comparable post-thaw outcomes. Although the CryoKit generated a different cooling curve and reached −80 °C in less time, these differences did not translate into measurable effects on sperm quality. In addition, the shorter cooling period increased processing throughput, allowing approximately three additional freezing runs per hour. Combined with its lower cost and ease of implementation, these characteristics offer practical advantages for laboratories and germplasm repositories.

The cooling curve is a critical factor influencing cryopreservation success, as inappropriate cooling rates can increase cryoinjury through intracellular ice formation and osmotic stress.^28^^,^^29^^,^^30^ While the computer-controlled freezer applies active, regulated cooling to follow a programmed profile, the CryoKit relies on passive cooling within a temperature gradient, resulting in a more curvilinear cooling trajectory. Despite these differences in cooling dynamics, no differences were observed in post-thaw sperm viability, forward motility, or twitching motility between the two methods. These findings suggest that both approaches provide cooling conditions sufficient to minimize cryoinjury and maintain sperm quality during cryopreservation.

Furthermore, many low-cost cryopreservation approaches fail to report essential parameters such as cooling curves, container geometry, and liquid nitrogen depth, undermining reproducibility and cross-study comparisons.^31^^,^^32^ The CryoKit mitigates these limitations by providing open-hardware files and assembly instructions that allow replication of device geometry and thermal behavior, and also enable community-driven adaptations and improvement. With 3D printers becoming more accessible,^32^ open-hardware devices are becoming accessible anywhere in the world by simply printing the file and following the instructions to cryopreserve any type of sample with access to different time from 5 to −80 °C configurations.

### Evaluation of gelatin capsules for sperm cryopreservation

The choice of container for cryopreservation is a critical step that goes beyond commercial availability. The type of container can influence the outcomes of processing and cryopreservation, as physical properties impact the cooling rate.^26^ Our results show that the time required for samples to reach −80 °C did not differ between samples cryopreserved in gelatin capsules or straws. Likewise, the post-thaw outcomes of cryopreserved samples were similar across treatments, suggesting that the capsule format, together with the 3D printed modular holder, was able to reproduce the thermal behavior of the French straw during cooling.

Although the capsule format requires a 3D printed modular holder to fit on the CryoKit, and this holder could be a potential source of added thermal mass, it was designed to reduce thermal mass as much as possible. These findings suggest that combination of the capsule and 3D printed modular holder reproduced French straw thermal behavior during cooling and post-thaw outcomes. It is possible to customize the Cryokit to hold gelatin capsules directly, thereby reducing thermal mass around the gelatin capsules, but that would introduce the challenge of transferring frozen gelatin capsules into the modular holding system while maintaining submersion in liquid nitrogen.

It is critical to measure, adjust, and report sperm concentration.^33^ Differences in concentration can have profound effects on cryopreservation, including cryoprotectant efficacy and fertilization success.^34^ In this study, cryopreservation reduced sperm viability relative to fresh samples, regardless of the container used or the thawing procedure. However, no differences were detected among the cryopreserved treatments, indicating that dilution during or after thawing did not further affect viability under the conditions tested. França et al.^20^ reported reduced post-thaw viability in South American catfish (*Rhamdia quelen*) sperm following dilution of thawed capsule samples. In contrast, no effect of dilution was observed in *X. laevis*, suggesting that the influence of post-thaw dilution varies among species and according to sample handling procedures. Although dilution has been associated with impaired membrane integrity and increased reactive oxygen species (ROS) production,^35^^,^^36^ these effects were not reflected in viability differences among cryopreserved treatments, indicating that additional damage attributable to dilution was not evident under the present conditions.

Dilution can influence sperm motility by altering the viscosity of the medium. A reduction in viscosity can decrease *X. laevis* sperm motility,^37^ and the physical pressure provided by a viscous medium may play an important role in promoting the switching of activated doublet microtubules.^38^ However, in the present study, no differences in progressive motility were observed among cryopreserved treatments, suggesting that the dilution conditions applied did not differentially affect this parameter. Fertilization capacity also did not differ significantly among cryopreserved treatments, and when sperm concentrations per egg number (fertilization dose) were standardized, all cryopreservation procedures evaluated here maintained functional competence for fertilization. Although no statistically significant differences were detected, the effect size was relatively high, influenced in part by the exclusion of one female from the analysis due to an *a priori* reason, as the eggs did not meet the minimum quality threshold required for inclusion. This exclusion should be taken into consideration when interpreting these results. Nevertheless, this variability does not alter the overall pattern observed in post-thaw sperm quality, as the absence of consistent differences in motility and related parameters supports functional equivalence among cryopreservation treatments.

Although these results indicate that dilution during thawing does not impair post-thaw sperm quality and fertilization in *X. laevis*, the effects of dilution should be assessed in other animal species, as it may be detrimental in species with naturally dilute sperm, such as frogs or other amphibians in which sperm can be collected via spermic urine, since these samples are typically more dilute than sperm obtained by dissecting the testes.^39^

Another challenge was that gelatin capsules dissolve in aqueous media, so conventional thawing by direct immersion in a water bath risks premature exposure of the sample to water, which can lead to contamination and sample degradation. To address this, dissolving the capsule directly in an extender for fish sperm was proposed.^20^ However, dilution could reduce sperm viability and fertilization capability of frog sperm. To overcome this limitation, the present study developed a dry-thawing technique using glove fingers cut and stretched over a plastic tube, ensuring effective recovery of the cryopreserved material without unintended dilution.

Regarding gelatin capsules, although they are hygroscopic and biodegradable, they showed stable structural integrity under liquid nitrogen storage in our experience, with no evidence of liquid nitrogen penetration. The main risk of rupture appears to be associated with abrupt temperature changes rather than long-term cryogenic storage. Nevertheless, further long-term studies are still required to confirm complete sterility and safety under extended storage conditions.

### Open hardware as a community tool

It is becoming increasingly evident that open hardware can provide a powerful community-level approach that can enable standardization across species.^26^ In this study, we expand this concept by demonstrating that open hardware is most effective when applied not as isolated devices, but as an integrated cryopreservation system. By combining the CryoKit platform,^14^ previously developed for low-cost controlled freezing, with gelatin capsule-based sample containment,^20^ we establish a unified and reproducible workflow that connects all stages of the process, including sample loading, freezing, storage, identification, handling, and thawing. This integration also addresses key practical limitations of capsule-based systems, such as instability during handling, lack of individual labeling, and risks associated with conventional thawing procedures, through the development of a modular holder and a dry-thawing method.

Together, these components form a complete and operational pipeline rather than independent technological elements. By improving usability while maintaining low cost and accessibility, this integrated framework strengthens the potential of open hardware to democratize cryopreservation technologies and support the development of standardized protocols for amphibian conservation in resource-limited settings. This can also support development of multidisciplinary collaborations empowering a new generation of technology-minded biologists (e.g., “technobiologists”) who can easily cross disciplines to solve big problems.

This study demonstrates a practical, low-cost open-hardware approach for amphibian sperm cryopreservation by combining 3D-printed devices with gelatin capsules. We provided a system for capsule handling and a dry-thawing technique. These innovations can be effectively integrated into captive breeding and reintroduction programs, offering promising new standardizable methods for amphibian conservation in resource-limited settings. By making cryopreservation more accessible, these approaches can help democratize the technology, strengthening global efforts to preserve the genetic diversity of threatened amphibian species.

### Limitations of the study

The fertilization analysis was based on a small effective sample size, as one female was excluded following an *a priori* egg-quality criterion, which reduces the statistical power of this specific endpoint and should be considered when interpreting the fertilization results. In addition, all trials were conducted using a single model species (*X. laevis*), so validation with additional amphibian species will be needed before the performance of the open-hardware platform can be generalized across taxa.

## Resource availability

### Lead contact

Requests for further information and resources should be directed to and will be fulfilled by the lead contact, Thaiza Rodrigues de Freitas (thaiza.rfreitas@gmail.com).

### Materials availability

All open-hardware files are publicly available through the NIH 3D Print Exchange.

### Data and code availability

• Data: All raw experimental data generated or analyzed during this study, including individual animal data, cooling curves, and results from all experiments, have been deposited in the GitHub repository (https://github.com/thaizarfreitas/Amphibian-open-hardware-cryopreservation-data.git) see key resources table.
• Code: This study did not generate original code.
• Other: The 3D design files for the capsule filling rack and modular holder are publicly available through the NIH 3D Print Exchange (https://3d.nih.gov/entries/3DPX-021806). The CryoKit open-hardware files used in this study are also available through the NIH 3D Print Exchange (https://3d.nih.gov/entries/3DPX-021194). See the key resources table for accession identifiers.

## Acknowledgments

This work was supported in part by the 10.13039/100000002National Institutes of Health Office of Research Infrastructure Programs (R24OD028443 and R24OD037797). Additional support was provided by the 10.13039/100005825National Institute of Food and Agriculture, United States Department of Agriculture (Hatch project LAB94420), the 10.13039/100000001National Science Foundation (NSF POSE 2229680), the LSU Veterinary School – ACRES program (grant: “Development of Practical On-site Cryopreservation Technologies for Critically Endangered Amphibians with the Axolotl (*Ambystoma mexicanum*) as a Model”), the 10.13039/501100002322Coordenação de Aperfeiçoamento de Pessoal de Nível Superior (CAPES, Brazil) (88887.877624/2023-00 to T.R.F.), and the 10.13039/501100003593National Council for Scientific and Technological Development (CNPq, Brazil) Research Fellowship Program (141423/2021-8 and 305387/2022-7). We gratefully acknowledge the staff at the National Xenopus Resource (NXR P40OD010997) for their resources and support. This manuscript was approved for publication by Louisiana State University Agricultural Center as number 2025-2087-40526.

## Author contributions

Writing – original draft, T.R.F.; visualization, T.R.F.; investigation T.R.F., E.H., N.S.T., and J.L.B.; data curation, T.R.F. and T.S.F.; conceptualization, T.R.F., D.P.S.J., T.S.F., J.C.K., and T.R.T.; project administration, T.R.F.; writing – review and editing, D.P.S.J., E.H., N.S.T., J.L.B., T.S.F., J.C.K., and T.R.T.; methodology, E.H., N.S.F., and J.L.B.; formal analysis, T.R.F. and T.S.F.; funding acquisition, J.C.K. and T.R.T.; resources, J.C.K. and T.R.T.; supervision, D.P.S.J. and T.R.T.

## Declaration of interests

T.S.F. and D.P.S.J. filed (September/2022) a patent (BR10202201781) application on the methodology using gelatin capsules for sperm cryopreservation described in the study. The remaining authors have no conflicts of interest that could be perceived as prejudicing the impartiality of the research reported.

## Declaration of generative AI and AI-assisted technologies in the writing process

The authors employed ChatGPT to help identify potential spelling mistakes and to check the consistency between in-text citations and the reference list. All content was subsequently reviewed and refined by the authors, who assume complete responsibility for the final published work.

## STAR★Methods

### Key resources table

**Table:** 

REAGENT or RESOURCE	SOURCE	IDENTIFIER
**Chemicals, peptides, and recombinant proteins**		

Tricaine methanesulfonate (MS-222)	Syndel	Cat#NC0872873
Sodium bicarbonate	Fisher Scientific	Cat#S233
Sodium chloride	Sigma-Aldrich	Cat#S9888
Potassium chloride	Thermo Scientific	Cat#011595.30
Magnesium sulfate	Fisher Scientific	Cat#M63
Calcium chloride dihydrate	Fisher Scientific	Cat#C79
HEPES	Thermo Scientific	Cat#329850500
Human chorionic gonadotropin (hCG)	Sigma-Aldrich	Cat#CG10
N,N-Dimethylformamide (DMF)	Sigma-Aldrich	Cat#D4551
Eosin Y	Sigma-Aldrich	Cat#230251
L-cysteine	Beantown Chemical	Cat#127565

**Deposited data**		

Raw experimental data	This paper; GitHub	https://github.com/thaizarfreitas/Amphibian-open-hardware-cryopreservation-data.git
3D Design Files (Capsule Rack & Modular Holder)	This paper; NIH 3D 3D Print Exchange	NIH 3D Print Exchange: 3DPX-021806
Positional Cooling Platform Device (CryoKit)	Hu et al., 2017; NIH 3D Print Exchange	NIH 3D Print Exchange: 21194/2

**Experimental models: Organisms/strains**		

Adult *Xenopus laevis* (wild type)	Aquatic Germplasm and Genetic Resources Center (AGGRC)	N/A

**Software and algorithms**		

Fusion 360 (CAD Software)	Autodesk Inc.	https://www.autodesk.com/products/fusion-360/personal
R (Statistical Computing)	The R Foundation	Version 4.2.2
RStudio (IDE for R)	Posit Software	Version 2022.07.01
GraphPad Prism (Graphing & Statistics)	GraphPad Software	Version 10.2.0
HOBOware (for thermocouple logger)	Onset Computer Corp.	https://www.onsetcomp.com/products/software

**Other**		

Gelatin Capsules (hard, size 03)	XPRS Nutra	N/A
0.25-mL French Straws (PVC)	IMV Technologies	Cat#005703
Polylactic Acid (PLA) Filament	ZYLtech Engineering	N/A
Creality Ender-3 S1 3D	PrinterCreality	N/A
Computer-Controlled Freezer (IceCube® 14M)	SY-LAB	N/A
4-Channel Thermocouple Logger (HOBO)	Onset Computer Corp.	Cat#UX120-014M
Neubauer Hemocytometer	Fisher Scientific	Cat#0267110
Makle chamber	Selfi-Medical Instruments ltd.	Cat#SM-363

### Experimental model and study participant details

#### Animal handling

Adult *Xenopus laevis* (wild type) were used in this study, comprising 3 females and 18 males, all approximately 2 years old. Animals were maintained in recirculating aquatic housing systems at 18 °C–19 °C under a 12 h:12 h light–dark photoperiod. The husbandry regimen included feeding commercial frog brittle (Nasco, Ft. Atkinson, WI) three times per week. Water quality was monitored weekly, maintaining the following parameters: ammonia 0–1.0 mg/L, nitrite 0–0.8 mg/L, nitrate 0–40 mg/L, alkalinity 80–300 mg/L, pH 7.0–8.5, and hardness 100–250 mg/L.

All procedures were conducted in accordance with the Institutional Animal Care and Use Committee (IACUC) of the Louisiana State University Agricultural Center (Protocol A2024-14). The study design incorporated both sexes; however, because the experimental objectives focused on male gamete cryopreservation, sex-associated differences were not evaluated. This should be considered a limitation regarding the broader generalizability of the findings.

### Method details

#### Capsule devices design, fabrication, and functionality testing

To facilitate the use of gelatin capsules, open hardware devices were developed. The devices were designed using computer-aided design (CAD) software (Fusion, Autodesk Inc., USA). Devices were prototyped by fused filament fabrication (FFF) 3D printing (Creality Ender-3 S1, Shenzhen, China; ∼US$200) using polylactic acid (PLA) filament (ZYLtech Engineering, Spring, TX). Preliminary functional testing was conducted to evaluate efficacy in handling and storing gelatin capsules. Empty gelatin capsules (hard-gelatin, size 03; XPRS Nutra, USA) were filled with 100 μL of distilled water to simulate the sample. The filled gelatin capsules were placed into the handling and storage device, and they were assessed for ease of handling, stability, and security of the gelatin capsules during manipulation, freezing, and overall efficiency in organizing capsules for storage.

#### Gamete collection

Males were euthanized by immersion in a 0.4% solution of tricaine methanesulfonate (MS-222), buffered with 0.4% sodium bicarbonate, for 20 min, followed by cervical transection to ensure death. Testes were then immediately dissected and carefully cleaned on absorbent paper to remove any residual blood and connective tissue.

For sperm collection, each testis was placed in a 1.5-mL microcentrifuge tube containing 400 μL of ice-cold 1.2× Marc’s Modified Ringer’s solution (MMR; 0.12 M NaCl, 0.0024 M KCl, 0.0132 M MgSO_4_, 0.0024 M CaCl_2_, 0.006 M HEPES; 250 mOsm/kg).^40^ The tissue was mechanically disrupted using a sterile 70-mm plastic pestle to release spermatozoa into the solution.^27^ The resulting suspension was used as the sperm source for subsequent procedures. Sperm concentration and motility was measured using a Neubauer hemocytometer (Optiphot-2 microscope, Nikon, 20-× objective lens). All sample concentrations were adjusted with 1.2 × MMR to 1.6 × 10^8^ sperm/mL or 3.2 × 10^8^ sperm/mL depending on the treatment. After adjusting concentration and estimating motility for each male, samples were pooled by mixing equal volumes of sperm suspension (1 mL) from three different males.

For egg collection, female *X. laevis* were induced to ovulate by subcutaneous injection of 5 IU/g body weight of human chorionic gonadotropin (hCG, Sigma-Aldrich) 12 to 18 h prior to collection.^27^ The females were held in individual containers overnight, and eggs were collected by gently massaging the abdomen. Expressed eggs were fertilized within 1 min.

#### Experimental design

##### Sperm cryopreservation with different cooling devices

The effectiveness of the 3D-printed *Positional Cooling Platform Device* (PCPD, known as ‘CryoKit’)^14^ was compared to a computer-controlled freezer (IceCube 14M, SY-LAB, Austria). The CryoKit (V2.4.7) included 14 3-D printed components, two pieces of Styrofoam to provide floatation, and nested Styrofoam boxes.^14^ Three sperm pools were randomly distributed into three treatments: (1) CryoKit; (2) computer-controlled freezer, and (3) fresh control. Samples were cryopreserved into 0.25-mL French straws.

#### Evaluation of gelatin capsules for sperm cryopreservation

Gelatin capsules were compared to 0.25-mL French straws (PVC; IMV Technologies, France) as containers for cryopreservation. Each gelatin capsule could hold as much as 200 μL of liquid. In addition, a novel dry thawing method for gelatin capsules (detailed in Section 2.5) was compared to a previously developed method in which capsules were thawed within the extender solution.^20^ To enable a balanced comparison of the cryopreservation containers, the experimental design included an additional treatment in which samples from straws were diluted after thawing (100 μL of cryopreserved sperm in a gelatin capsule or straw diluted in 1 mL of extender). Three sperm pools were randomly distributed into five treatments: (1) gelatin capsule - dry-thawing; (2) gelatin capsule - dilution thawing; (3) straw; (4) straw – diluted after thawing, and (5) fresh control.

#### Quality evaluation

Quality measures were evaluated for each individual male (*n* = 9) except for fertilization which was evaluated per pool (*n* = 3). For the cooling device experiment, males had a mean body weight of 65.81 ± 8.53 g, sperm concentration of 3.75 × 10^8^ ± 1.45 × 10^8^ sperm/mL, forward motility of 39.33 ± 9.66%, twitching motility of 18.67 ± 8.28%, and viability of 67.18 ± 4.06%. For the gelatin capsule experiment, males had a mean body weight of 59.99 ± 10.08 g, sperm concentration of 3.60 × 10^8^ ± 1.69 × 10^8^ sperm/mL, forward motility of 38.89 ± 9.66%, twitching motility of 22.00 ± 9.39%, and viability of 60.72 ± 8.86%.

#### Viability

Sperm membrane integrity was evaluated by a dye exclusion assay as an estimator of viability.^41^ For each sample, 5 μL of 0.4% eosin Y (Diluted in 1.2-× MMR; Sigma-Aldrich) was added to 10 μL of sperm. A total of 200 spermatozoa were classified by use of light microscopy with a 40-× objective (400-× total magnification, Microphot-SA, Nikon). Sperm were considered to have damaged membranes if they exhibited a pinkish color, which indicated that dye had been able to enter the cell because the plasma membrane was compromised. Sperm that remained non-stained were considered to have intact membranes.

#### Motility

To evaluate sperm motility, sperm samples were activated using a 1:1 dilution with deionized (DI) water. Ten microliters of activated sperm were immediately placed on a Makler counting chamber. Motility was assessed visually for 10 s after activation, by use of a light microscope with a 40-× objective (400-× total magnification, Microphot-SA, Nikon). A total of 100 sperm cells were evaluated and categorized into three groups: (1) moving forward (MF); (2) exhibiting twitching movements (TM) non-progressive movement,^27^ and (3) non-motile.

#### *In vitro* fertilization

For the *in vitro* fertilization (IVF) procedure ∼50 eggs were collected and placed into the center of a 100 × 25 mm Petri dish. Sperm were activated by 1:1 dilution with 0.1-× MMR (25 μL of sperm: 25 μL 0.1-× MMR; 50 μL total), then added and gently mixed with the eggs using a modified microbiology plastic inoculation loop.^27^ For the treatments diluted after thawing, sperm were activated using the same dilution approach (125 μL sperm +125 μL MMR; 250 μL total) and applied to the eggs to maintain a consistent concentration (∼2 × 10^6^ sperm/dish).

After mixing, the sperm and eggs were left undisturbed for 5 min to allow fertilization.^27^ The dish was flooded with 0.1-× MMR solution. Embryos were incubated in 100 × 25 mm Petri dish at room temperature (21 °C–23 °C) for 48 h. The fertilization procedure was performed in duplicate for each treatment.

To facilitate observation and removal of unfertilized embryos, a 2% L-cysteine solution (diluted in deionized water, pH 8.5) was used to remove egg gel. One hr post-fertilization (HPF), eggs were immersed in cysteine and gently agitated until the gel dissolved (<7 min). The embryos were rinsed three times with 0.1-× MMR. At 24 HPF, embryos that had reached the neurula to late neurula stages were retained in the Petri dishes, while undeveloped oocytes were removed before replacing the 0.1-× MMR. At 48 HPF, fertilization capacity was reassessed by identifying embryos that had developed to approximately NF stage 40 (early swimming tadpole stage), characterized by clear spontaneous movement, followed by replacement of the 0.1-× MMR. Only females with fertilization capacity rates of at least 60% when using fresh sperm (control) were retained for analysis. This minimum threshold was established *a priori*, before fertilization assays, as a criterion to determine egg quality and ensure that only females producing eggs with adequate fertilization potential were included. One female with 0% fertilization was excluded from the analyses.

#### Cooling time calculations

Because the cooling curves for cryopreservation with open-hardware devices were not linear, we chose to report cooling time rather than average cooling rate. Reporting the time required to reach −80 °C provides a simple and practical metric that allows users to verify device performance by directly observing the time needed to reach the target temperature, without requiring cooling-rate calculations. Time from 5 °C (corresponding to the temperature at the end of the equilibration time) to −80 °C was recorded (at 1-s intervals) using a 4-channel thermocouple logger (HOBO, Onset Computer Corp., Bourne, MA). Each cryopreservation container type (straw or gelatin capsule) was filled with 100 μL of cryoprotectant solution without sperm sample, and a thermocouple probe was positioned in the cryoprotectant solution; a small hole was made in the top of the capsule to accommodate the probe wire.

For the computer-controlled freezer, the minimum sample temperature was −75.7 °C; thus, the cooling rate was converted as cooling time from 5 to −80 °C.

#### Cryopreservation

Samples were frozen at 1.6 × 10^8^ sperm/mL except for treatments requiring dilution during thawing, which were frozen at a double concentration (3.2 × 10^8^ sperm/mL). Samples were mixed in a 1:1 ratio with a cryoprotectant solution of 20% N,N dimethylformamide (DMF) in 1.2-× MMR, yielding a final concentration of 10% DMF.^27^ For cryopreservation in gelatin capsules, the samples were equilibrated in 1.5-mL microcentrifuge tubes on ice for 10 min. The sample was loaded into the capsule 1 min before the equilibration period ended, and each capsule contained 100 μL of the sample. For cryopreservation in straws, 100 μL aliquots were loaded and similarly equilibrated on ice for 10 min.

Samples frozen in the computer-controlled freezer (IceCube) were cooled at a programmed rate of −20 °C/min (∼5 min to achieve −80 °C). The CryoKit was set up with the platform positioned 19 mm above the liquid nitrogen (Configuration in the manual - 1: top rack + A: polystyrene float), at an ambient temperature of −167 °C, to achieve a time from 5 to −80 °C of 4–5 min (cooling rate of 20 °C–25 °C/min). During CryoKit cooling, a thermocouple was inserted into one of the straws and gelatin capsules to monitor temperature until the samples reached −80 °C. At −80 °C all samples were plunged into liquid nitrogen for storage. Samples were stored for 7 days before thawing.

#### Thawing

**Figure 8:**
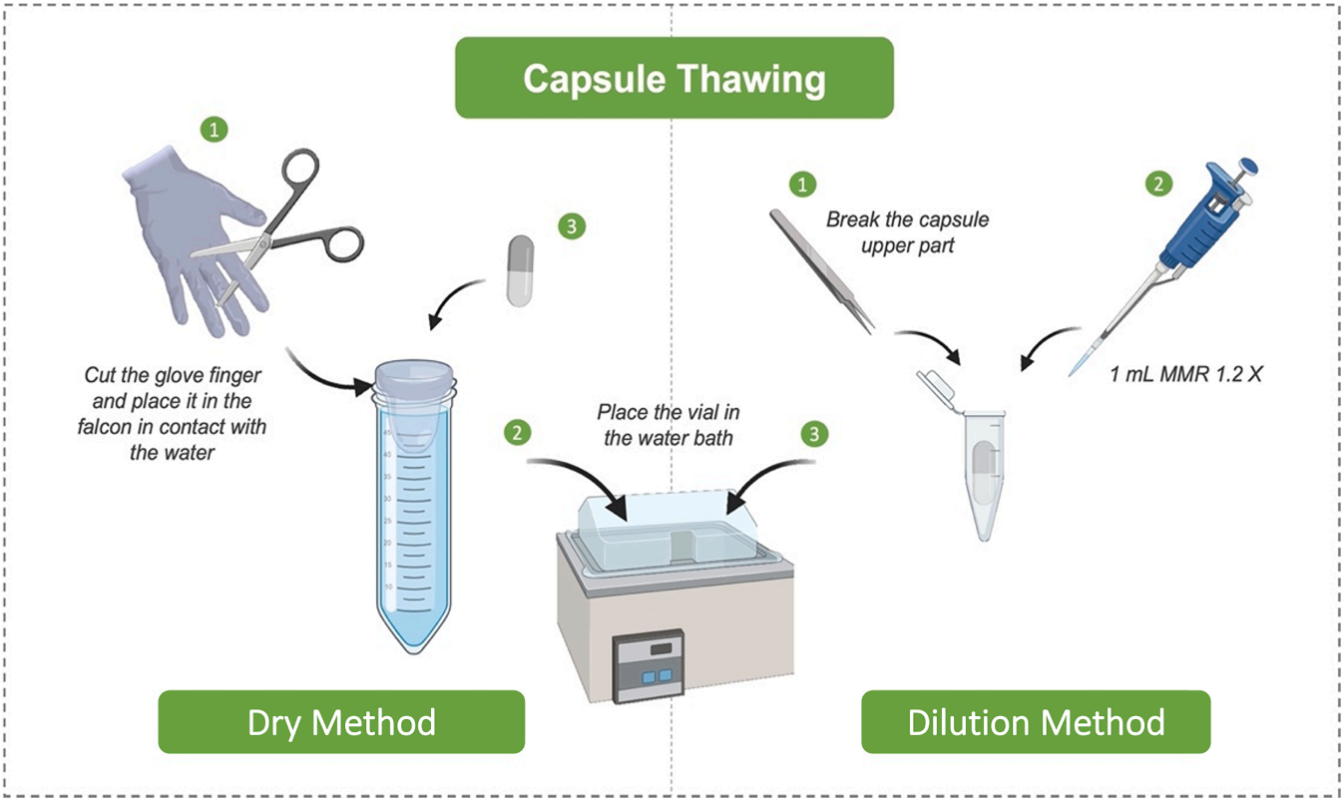
Gelatin capsule thawing methods The numbers (1, 2, and 3) on each image indicate sequential steps for the (left) dry and (right) dilution thawing methods.

To address the issues of capsule dissolution and possible water ingress during immersion in a water bath, as well as over-dilution of the sample caused by adding the extender inside the capsule during thawing (original methodology^20^), two thawing protocols were tested. For the non-diluted capsule treatment, a dry-thawing method was developed using a custom glove-finger pouch. A pouch was created by cutting a 2-cm-long finger segment from a nitrile glove (glove thickness 0.14 mm; Fisherbrand, Pittsburgh, PA). A 50-mL conical tube (Falcon, Corning Inc., Corning, NY) was filled with 50 mL of extender and pre-equilibrated in a 60 °C water bath. The glove-finger pouch was stretched securely over the mouth of the tube by fitting it around the tube threads. The closed end of the pouch was pushed into the tube so that it extended downward and contacted the extender inside, forming a suspended “nest” for the capsule while preventing direct contact between the capsule and the extender (Figure 8, left). The tube was placed upright in a rack within the 60 °C water bath and capped to maintain the internal temperature. For thawing, a frozen gelatin capsule was rapidly placed into the pouch, the tube was immediately sealed, and the capsule was thawed for 45 s.

The diluted capsule treatment was thawed following the method of França et al.^20^ Briefly, each capsule was transferred to a 1.5-mL microcentrifuge tube, and the upper half was broken with tweezers before adding 1 mL of 22 °C 1.2-× MMR solution (Figure 8, right). The tube was placed in a water bath set to 40 °C for thawing for 20 s with gentle mixing using a pipette. After thawing, samples were transferred to a 1.5-mL microcentrifuge tube and placed on ice until further use.

Samples cryopreserved in straws were thawed by immersion in a 40 °C water bath for 5 s. The ends of the straw were cut and the sample released into a 1.5-mL microcentrifuge tube. For the diluted straw treatment, 1 mL of extender was added to the sample immediately after thawing.

### Quantification and statistical analysis

The data are presented as mean ± SD. Normality (Shapiro–Wilk test) and homogeneity of variances (O’Neill & Mathews test) were verified. In addition, QQ plot and residual plot were visually inspected when appropriate to verify compliance with the assumptions of parametric analyses. The data were considered to adequately meet these assumptions (*p* > 0.05), and no data points were excluded from the analyses. Each sperm pool was considered an experimental replicate and included as a blocking factor in the statistical analyses. Data were analyzed using one-way ANOVA followed by Tukey’s *post-hoc* test. The times from 5 to −80 °C were evaluated by unpaired Student’s t test. Statistical significance was set at *p* < 0.05. Effect sizes were calculated using Hedges’ g to quantify the magnitude of differences between treatments. Values of Hedges’ g around 0 indicate negligible or no effect, values around 0.2 indicate small effects, around 0.5 indicate medium effects, and values ≥0.8 indicate large effects. Fertilization data from one female were excluded from the statistical analysis based on an *a priori* established criterion, as fertilization rates were consistently close to zero across all treatments, including the fresh sperm control. Analyses were performed using R (version 2025.09.02) and Graphs were generated using GraphPad Prism (version 10.2.0).
